# Electrical Properties and Interfacial Issues of HfO_2_/Ge MIS Capacitors Characterized by the Thickness of La_2_O_3_ Interlayer

**DOI:** 10.3390/nano9050697

**Published:** 2019-05-04

**Authors:** Lu Zhao, Hongxia Liu, Xing Wang, Yongte Wang, Shulong Wang

**Affiliations:** Key Laboratory for Wide Band Gap Semiconductor Materials and Devices of Education, School of Microelectronics, Xidian University, Xi’an 710071, China; lzhaoxd@163.com (L.Z.); xwangsme@xidian.edu.cn (X.W.); mikewyt@163.com (Y.W.); slwang@xidian.edu.cn (S.W.)

**Keywords:** Ge surface engineering, La_2_O_3_ passivation layer, atomic layer deposition, electrical properties

## Abstract

Effects of the La_2_O_3_ passivation layer thickness on the interfacial properties of high-k/Ge interface are investigated systematically. In a very thin range (0~15 cycles), the increase of La_2_O_3_ passivation layer deposition cycles improves the surface smoothness of HfO_2_/Ge structures. The capacitance-voltage (*C-V*) characteristics show that the thickness of La_2_O_3_ passivation layer can affect the shift of flat band voltage (*V*_FB_), hysteretic behaviors, and the shapes of the dual-swept *C-V* curves. Moreover, significant improvements in the gate leakage current and breakdown characteristics are also achieved with the increase of La_2_O_3_ interlayer thickness.

## 1. Introduction

Along with the continuing scaling of complementary metal oxide semiconductor (CMOS) technology according to Moore’s Law, insulation oxides with higher permittivity have been introduced to replace SiO_2_ for acceptable gate leakage current density and low power consumption [1]. However, other than reducing feature size, technological progress to gain high-speed operation is also needed with the development of integrated circuit (IC) technology. Considering this, high mobility semiconductors have been considered as alternative channel materials for obtaining high drive current. Among the high mobility semiconductors, germanium (Ge) is compatible with standard Si CMOS process, and both the electron and hole bulk mobility are higher than those of Si substrate [2,3]. Therefore, Ge has been regarded as one of the most promising alternative channel materials. Unfortunately, different from the perfect interfacial properties of SiO_2_/Si interface, the poor thermal stability of GeO_2_/Ge interface results in the deterioration of interfacial properties to a great extent [4]. When most gate dielectric materials deposited on the unpassivated Ge substrate, the generation of unstable Ge oxides is unavoidable during the high temperature post-deposition annealing (PDA) process, and the decomposition or desorption from GeO_2_ to volatile GeO would bring in a great deal of defects. Consequently, to get acceptable electrical properties of high-k/Ge structures, a suitable surface passivation treatment is a necessary technical issue prior to the deposition of high-k gate dielectrics on Ge substrates [5].

Owing to the much better thermodynamic stability than that of GeO_2_/Ge, fewer interface traps exist at the interface between La-based oxides and Ge substrates [6]. Consequently, La-based oxides have been considered as one kind of the alternative gate dielectric materials deposited on Ge to realize good electrical performance of Ge-based metal-insulator-semiconductor (MIS) devices [7,8]. However, the hygroscopicity of La_2_O_3_ layer limits its application as gate insulators [9]. In a previous study, we have proved that a ~2 nm La_2_O_3_ interlayer could effectively improve the electrical performance of Ge MIS devices, resulting in more than one order of magnitude decrease in the gate leakage current density [10]. In this work, a further investigation was performed on the impacts of La_2_O_3_ passivation layer thickness on the interfacial properties of HfO_2_ insulators and Ge substrates.

## 2. Materials and Methods

La_2_O_3_ and HfO_2_ were deposited on Sb-doped n-type Ge (100) wafers with a doping concentration of 1 × 10^16^ cm^−3^ by atomic layer deposition (ALD) method (R-150, Picosun, Espoo, Finland). Prior to the deposition, all the wafers were treated using acetone and alcohol, followed by dipping into HF (2%) and deionized water by cyclic cleaning for 5 times to remove the defective native Ge oxide. Tris(isopropyl-cyclopentadienyl) lanthanum (La(^i-^PrCp)_3_) and tetrakis (ethylmethylamino) hafnium (TEMAH) were used as La and Hf precursor, while H_2_O was used as oxidant with reference to the reported literatures [11,12]. Under the chamber temperature of 300 °C, La_2_O_3_ layers were deposited by alternately introducing La(^i-^PrCp)_3_ and H_2_O precursors to the reactor chamber using high purity N_2_ (>99.999%) as the carrier gas with a typical ALD growth cycle of 0.3 s La(^i-^PrCp)_3_ pulse/4 s N_2_ purge/0.3 s H_2_O pulse/9 s N_2_ purge. Using these process parameters, the steady-state growth rate of La_2_O_3_ was approximately 0.85 Å/cycle. For an ALD growth cycle of HfO_2_, TEMAH was pulsed into the chamber by carrier gas for 0.3 s with a 8 s N_2_ purge, and then H_2_O was pulsed for 0.1 s followed by a 8 s N_2_ purge. As a result, a stable growth rate of 0.75 Å/cycle was obtained for HfO_2_. The thickness of the La_2_O_3_ passivation layer was tuned by varying the number of ALD cycles, and the cycle numbers of 5, 10, and 15 cycles were chosen. Then ~6 nm HfO_2_ was deposited as the gate insulator materials after the deposition of La_2_O_3_ passivation layer. For comparison, the sample with only ~6 nm HfO_2_ as the gate oxide was also fabricated as a control sample. After the deposition of gate insulators, rapid thermal annealing (RTA) was carried out at 400 °C for 90 s in N_2_ ambient for all the wafers. For simplicity, the control sample is assigned as S1, and samples with 5, 10, and 15 ALD cycles of La_2_O_3_ passivation layers are assigned as S2, S3, and S4, respectively.

The physical thickness of the deposited films was optically measured using the Woollam M2000U spectroscopic ellipsometry (SE, Woollam Co. Inc., Lincoln, NE, USA), and the ellipsometry data were fitted using a Gen-Osc mode consisting of Gaussian and Tauc-Lorentz oscillators. The chemical bonding states of the interfaces between the deposited films and Ge substrates were examined by X-ray photoelectron spectroscopy (XPS, Axis Ultra DLD, Kratos Analytical, UK) measurements. Cross-sectional high-resolution transmission electron microscopy (HRTEM, TECNAI F20 system, ThermoFisher Scientific, Waltham, MA, USA) and energy dispersive X-ray spectroscopy (EDX) line scan measurements were performed to observe the microstructures and atomic compositions of the deposited films. The surface morphology of the films was monitored using atomic force microscopy (AFM, Dimension 3100, Veeco Digital Instruments by Bruker, Billerica, MA, USA) in tapping mode. The electrical properties of the HfO_2_/La_2_O_3_ gate stacks were evaluated using MIS capacitor structures. MIS capacitors were fabricated by ion-beam etching (IBE, IBE-A-150, Beijing Chuangshi Weina Technology Co., Ltd., Beijing, China) of e-beam evaporated 150 nm Al through a shadow mask with a diameter of 300 µm as the top gate electrode and 100 nm Al as the back ohmic contact. Then, the electrical properties of the fabricated MIS capacitors were measured using an Agilent B1500A parameter analyzer (Santa Clara, CA, USA).

## 3. Results and Discussion

### 3.1. Chemical Bonding States of the Interfaces between the Deposited Films and Ge Substrates

**Figure 1:**
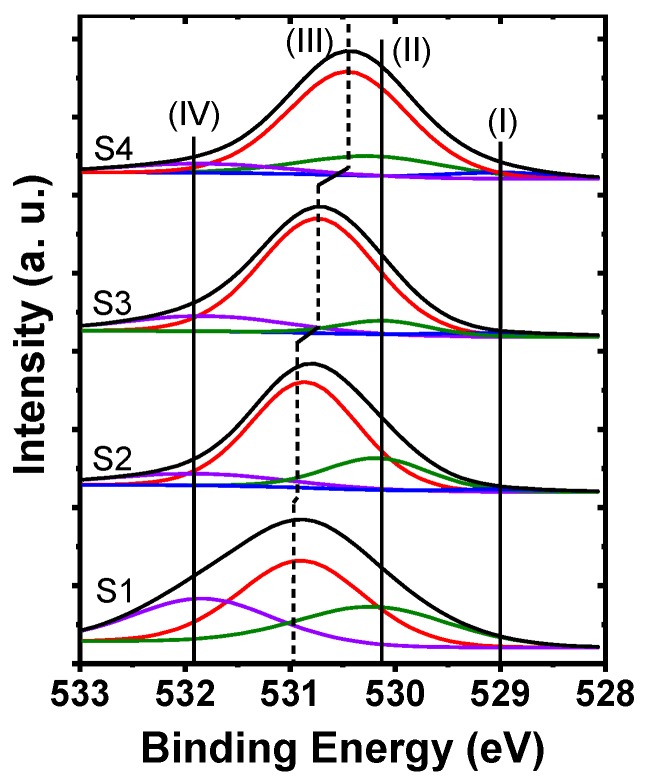
O 1s XPS spectra of HfO_2_ film and HfO_2_/La_2_O_3_ gate stacks deposited on Ge substrates.

The chemical bonding states near the interface of HfO_2_ and HfO_2_/La_2_O_3_ gate stacks deposited on Ge substrate were investigated by XPS measurements. Prior to the measurements, the wafers S1~S4 were etched by Ar^+^ ion beam bombardment for 25 s, 30 s, 30 s, and 35 s, respectively, under the etch rate of ~0.20 nm/s to remove the influence of the surface impurities and to analyze the chemical bonding states in the interfacial region. The XPS data were calibrated by setting the C 1s peak originated from the carbon impurities in the spectra at 284.6 eV for all the wafers. Figure 1 shows the variations of the O 1s XPS spectra for the HfO_2_ film and HfO_2_/La_2_O_3_ gate stacks deposited on Ge substrates. For the control sample (S1), the O 1s spectra consist of three Gaussian–Lorentzian line shape peaks. These peaks are located at 530.2, 531.0, and 531.9 eV, corresponding to the chemical bonding states of Hf–O–Hf, Hf–O–Ge, and Ge–O–Ge, respectively [13,14]. The existence of Hf–O–Ge and Ge–O–Ge peaks indicate that HfGeO_x_ and GeO_x_ were generated at the HfO_2_/Ge interface during the deposition of HfO_2_ and post-deposition annealing (PDA) process. As for the HfO_2_/La_2_O_3_/Ge structures, the O 1s core level spectra are deconvoluted into four peaks, three of which are at 529.0 (I), 530.2 (II), and 531.9 (IV) eV. These peaks correspond to the chemical bonds of La–O–La, Hf–O–Hf, and Ge–O–Ge, respectively [15]. The other peak (III) at around 530.9 eV originates from the Hf–O–Ge and/or La–O–Ge bonding states, indicating the generation of germanate (LaGeO_x_ and HfGeO_x_) compound at the interface of HfO_2_/La_2_O_3_ gate stacks and Ge substrates. It is worth noting that the position of peak (III) shifts to lower binding energies from sample S1 to S4. This phenomenon is attributed to the transformation from Hf-rich to La-rich interfacial layer (IL) with the increase of La_2_O_3_ passivation layer thickness, owing to the stronger reaction between La_2_O_3_ and outdiffused Ge atoms than that of HfO_2_ with outdiffused Ge atoms [16,17].

**Figure 2:**
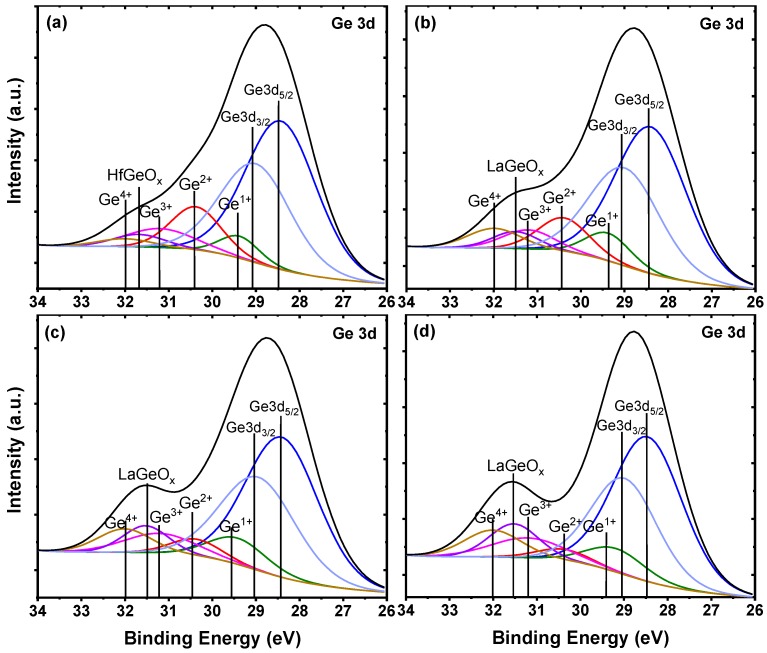
Ge 3d XPS spectra of the deposited gate stacks with different ALD cycles of La_2_O_3_ passivation layer on Ge substrates. (**a**) The control sample; (**b**) 5 ALD cycles of La_2_O_3_ passivation layer; (**c**) 10 ALD cycles of La_2_O_3_ passivation layer; and (**d**) 15 ALD cycles of La_2_O_3_ passivation layer.

To further discuss the variation tendency of Ge oxides at high k dielectrics/Ge interfaces with the increase of La_2_O_3_ passivation layer thickness, investigations on the Ge 3d XPS spectra are shown in Figure 2. A substrate peak (Ge 3d^0^) located at ~28.6 eV with additional peaks at higher binding energies in relation to the IL (consisting of Ge oxides and germanate) could be observed in all spectra. The Ge 3d^0^ substrate peak is fitted with a doublet of Ge 3d_5/2_ and Ge 3d_3/2_ with spin-orbit splitting of 0.6 eV and intensity ratio of 3:2, respectively [18]. The Ge oxides (GeO*_x_*) consist of four peaks (Ge^1+^, Ge^2+^, Ge^3+^, Ge^4+^), which are at higher binding energy respect to the Ge 3d^0^ with energy shifts of 0.8, 1.8, 2.6, and 3.4 eV, respectively [19]. Compared with the control sample shown in Figure 2a, the intensity of the Ge^2+^(GeO) peak decreases with the increase of La_2_O_3_ passivation layer thickness, while the intensity of the LaGeO*_x_* peak shows an increasing trend. Consequently, with the increase of La_2_O_3_ passivation layer thickness, the variation tendency of the interfacial components extracted from the Ge 3d XPS spectra is consistent with the results extracted from the O 1s XPS spectra.

### 3.2. Microstructures of the Deposited Films on Ge Substrates

**Figure 3:**
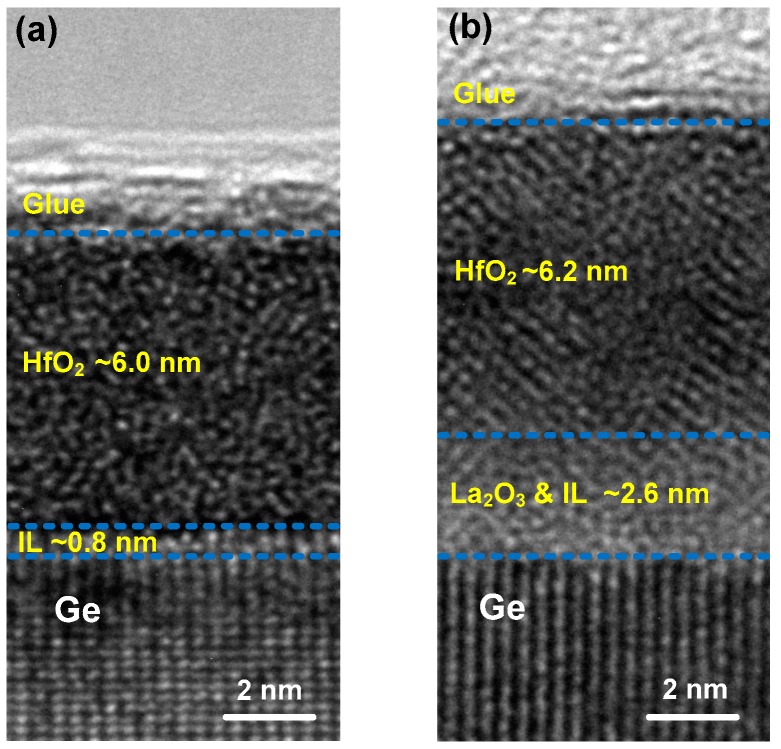
Cross-sectional HRTEM images showing the interfaces with Ge substrates for (**a**) the control sample; (**b**) sample S4 with 15 ALD cycles of La_2_O_3_ passivation layer.

**Figure 4:**
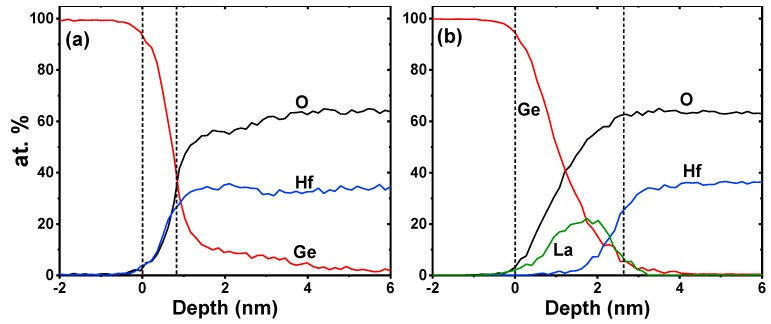
EDX profiles near the interfaces between the deposited films and Ge substrates. (**a**) the control sample; (**b**) sample S4 with 15 ALD cycles of La_2_O_3_ passivation layer.

Additional microstructure information of the deposited films is provided by cross-sectional HRTEM analysis as shown in Figure 3. For the control sample without La_2_O_3_ passivation layer, the HfO_2_ layer exhibits an amorphous structure as no nanometer-sized crystal or long-range ordered crystal region is observed. However, in sample S4 with 15 ALD cycles of La_2_O_3_ passivation layer (as shown in Figure 3b), nanometer-sized crystals could be observed in the HfO_2_ layer, indicating the incomplete crystallization state of the HfO_2_ film. It is noteworthy that upon the same RTA condition at 400 °C for 90 s in N_2_ ambient, the HfO_2_ films in sample S1 and S4 show different crystalline characteristics. This difference could be attributed to the RTA-induced Ge diffusion from the substrate into the HfO_2_ layer as shown in the EDX profiles near the interfaces between the deposited films and Ge substrates (Figure 4). The EDX data were dealt with normalization method after eliminating the influence of impurity elements, and the depth values were calibrated by HRTEM results. During the RTA process, in sample S1 as shown in Figure 4a, the substrate Ge atoms would diffuse easily to the upper HfO_2_ layer and react with the oxygen nearby the HfO_2_/Ge interface, which would prevent the formation of crystalline HfO_2_ precipitates [20] and cause the formation of IL (~0.8 nm) consisting of HfGeO*_x_* and GeO*_x_*, as analyzed in the above XPS results. As for the HfO_2_/La_2_O_3_/Ge structure in sample S4 (Figure 4b), due to the high affinity of La_2_O_3_ for Ge atoms [21], the out-diffused Ge atoms would react with La_2_O_3_ passivation layer, leading to the spontaneous formation of stable LaGeO*_x_* IL. As a result, few Ge atoms would diffuse into the HfO_2_ layer, and the crystallization phenomenon occurs after the RTA treatment.

### 3.3. Surface Morphology of HfO_2_ and HfO_2_/La_2_O_3_ Gate Stacks on Ge Substrates

**Figure 5:**
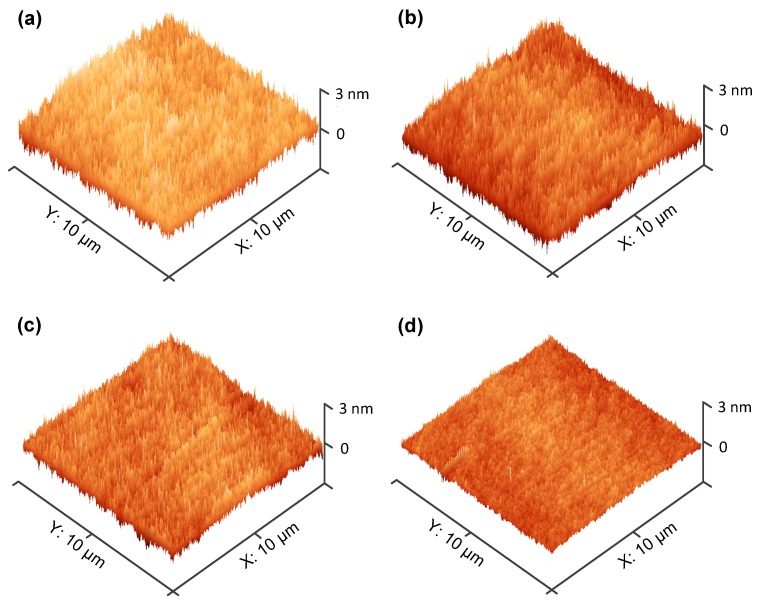
Three-dimensional AFM images of the deposited gate stacks with different ALD cycles of La_2_O_3_ passivation layer on Ge substrates. (**a**) The control sample; (**b**) 5 ALD cycles of La_2_O_3_ passivation layer; (**c**) 10 ALD cycles of La_2_O_3_ passivation layer; and (**d**) 15 ALD cycles of La_2_O_3_ passivation layer.

The AFM images of HfO_2_/La_2_O_3_ gate stacks on Ge substrates with 0, 5, 10, and 15 ALD cycles of La_2_O_3_ interfacial passivation layer are shown in Figure 5. The scan size of each AFM image was set as 10 × 10 µm^2^. The AFM images were captured from the AFM data by the software named Gwyddion. The root mean square (RMS) roughness of samples S1~S4 is extracted to be 0.63, 0.51, 0.28, and 0.25 nm, respectively. The small RMS roughness values of all the samples illustrate the smooth and crack-free surfaces of the deposited HfO_2_/La_2_O_3_ gate stacks, indicating that ALD is a good deposition method for high-k dielectric films and stacks. On the basis of the smooth surfaces, a decrease in the RMS roughness values is observed after inserting a La_2_O_3_ interfacial passivation layer, and the RMS roughness values decrease with the increase of La_2_O_3_ layer thickness. It has been reported that the outdiffused Ge atoms from substrates and the desorption of volatile species GeO during the high temperature annealing process could bring in large roughness [22]. After inserting La_2_O_3_ interfacial passivation layer, the stable LaGeO_x_ layer could suppress the formation of unstable GeO_x_ layer, which contributes to the decrease of the RMS roughness value. With the increase of La_2_O_3_ thickness, more La_2_O_3_ reacts with the outdiffused Ge atoms and unstable Ge oxides, which effectively suppresses desorption from GeO_2_ to volatile GeO, resulting in a smoother surface. It is found that when the thickness of La_2_O_3_ layer increase above 15 cycles, the variation in the RMS roughness values becomes negligible. The variation tendency of RMS roughness illustrates that 15 ALD cycles of La_2_O_3_ layer are thick enough to improve the surface smoothness for HfO_2_/Ge structures.

### 3.4. Electrical Performance of Al/HfO_2_/Ge and Al/HfO_2_/La_2_O_3_/Ge MIS Capacitors

**Figure 6:**
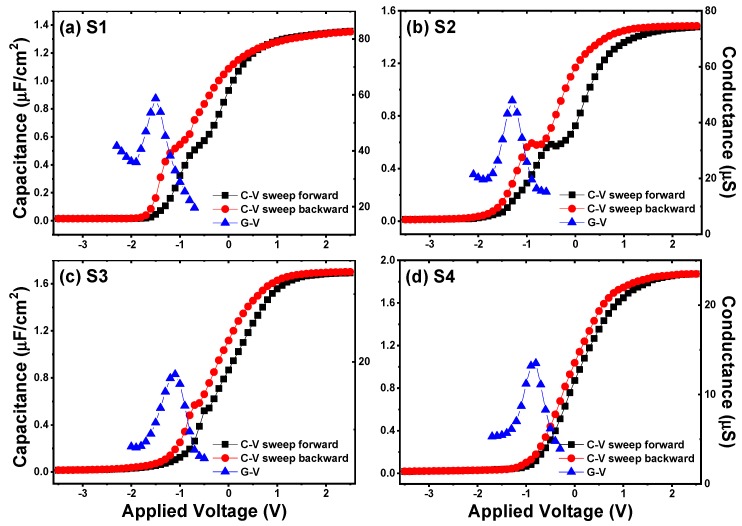
*C-V* curves and *G-V* characteristics of the fabricated Al/HfO_2_/La_2_O_3_/Ge MIS capacitors with different ALD cycles of La_2_O_3_ passivation layer. (**a**) The control sample; (**b**) 5 ALD cycles of La_2_O_3_ passivation layer; (**c**) 10 ALD cycles of La_2_O_3_ passivation layer; and (**d**) 15 ALD cycles of La_2_O_3_ passivation layer. The curves were measured at the frequency of 100 kHz.

The capacitance-voltage (*C-V*) and conductance-voltage (*G-V*) characteristics of the fabricated Al/HfO_2_/n-Ge and Al/HfO_2_/La_2_O_3_/n-Ge MIS capacitors are shown in Figure 6. Dual-swept *C-V* curves were obtained by sweeping from inversion to accumulation (forward) and sweeping back (backward) at the frequency of 100 kHz. *G-V* curves were obtained simultaneously with the *C-V* curves measured forward. To quantitatively characterize the effects of La_2_O_3_ passivation layer thickness on electrical performance of HfO_2_/Ge MOS structures, some parameters are extracted from the dual-swept *C-V* curves. The accumulation capacitance (*C*_ac_) values for gate stacks S1~S4 are obtained to be 1.35, 1.48, 1.69, and 1.87 μF/cm^2^, respectively. Then, the oxide capacitance (*C*_ox_) and dielectric constant values of gate stacks could be calculated following the equations [23,24]:

(1) $$C_{\mathrm{ox}}=C_{\mathrm{ac}}[1+{\left(\frac{G_{\mathrm{ac}}}{\omega C_{\mathrm{ac}}}\right)}^{2}]$$

(2) $$CET=\frac{\varepsilon_{0}\varepsilon_{{\mathrm{SiO}}_{2}}A}{C_{\mathrm{ox}}}$$

(3) $$k=\frac{\varepsilon_{{\mathrm{SiO}}_{2}}t_{\mathrm{ox}}}{CET}$$

where *C*_ac_ is the capacitance value at accumulation region, *G*_ac_ is the conductance corresponding to the accumulation region of the *C-V* curves, ω is the angular frequency, *C*_ox_ is the oxide capacitance of gate stacks, *A* is the area of the top electrode, *CET* is the capacitance equivalent thickness of deposited gate stacks (including IL), t_ox_ is the measured thickness of deposited gate stacks (including IL), ε_0_ and ε_SiO2_ are the permittivity values of vacuum and SiO_2_, respectively. The physical thickness of gate stacks (including IL) in S1, S2, S3, and S4 is optically measured to be 6.71, 7.89, 8.26, and 8.95 nm, respectively. The corresponding 95% confidence interval for the average thickness of the deposited films was shown in Table S1 in the Supplementary Materials to evaluate the discrete degree of thickness testing data. Therefore, according to Equations (1)–(3), the effective *k* values of S1~S4 are achieved to be 10.69, 13.43, 15.97, and 18.46, respectively. It is found that the dielectric constant value of HfO_2_/La_2_O_3_ gate stacks increases with the increase of La_2_O_3_ layer thickness. The improvement on the value of dielectric constant for the gate stacks is attributed to the effective suppression of Ge atoms outdiffusion benefiting from the generation of stable LaGeO*_x_*, since the *k* value of La_2_O_3_ is much higher than that of GeO*_x_* [25]. In addition, as analyzed in the EDX profiles, the HfO_2_ quality is improved by La_2_O_3_ passivation, which should be the other cause that increases the dielectric constant of the deposited films.

**Figure 7:**
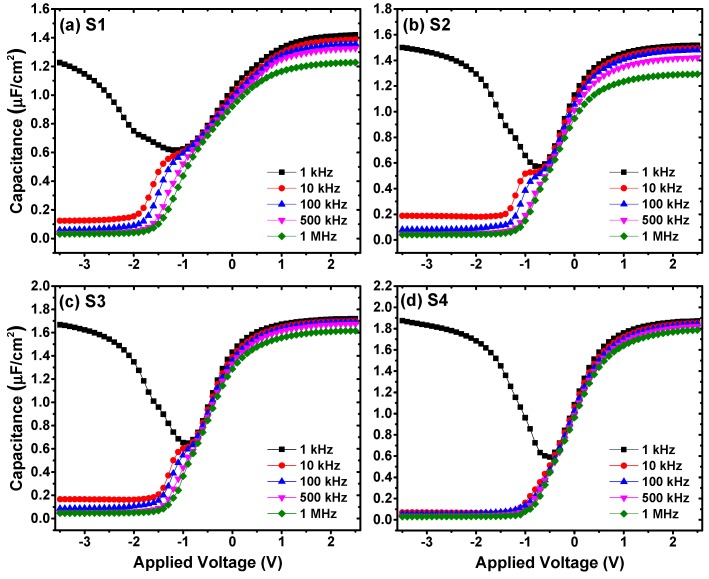
Multi-frequency *C-V* characteristics measured of the fabricated Al/HfO_2_/La_2_O_3_/Ge MIS capacitors with different ALD cycles of La_2_O_3_ passivation layer. (**a**) The control sample; (**b**) 5 ALD cycles of La_2_O_3_ passivation layer; (**c**) 10 ALD cycles of La_2_O_3_ passivation layer; and (**d**) 15 ALD cycles of La_2_O_3_ passivation layer.

**Figure 8:**
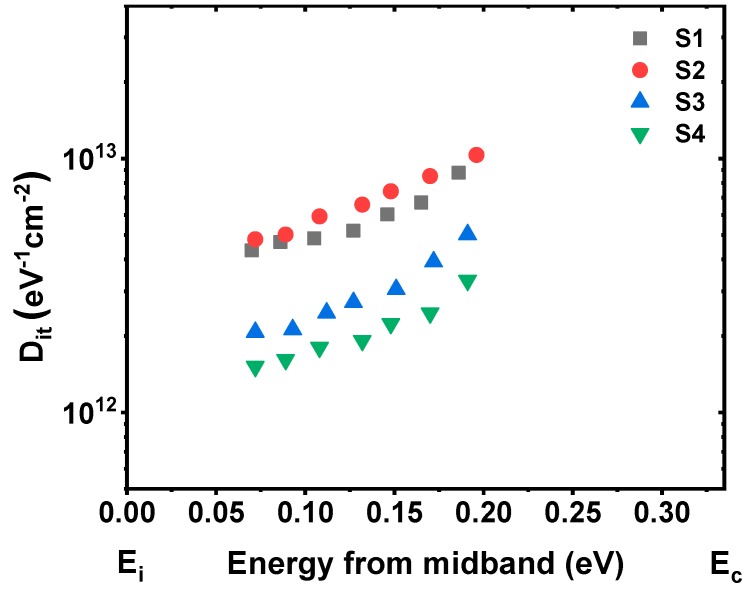
Energy distribution of the interface states at the interface between the deposited films and Ge substrates.

Moreover, as shown in Figure 6, varying degrees of anomalous humps in the weak inversion region of *C-V* curves could be observed. Compared with the control sample (Figure 6a), the anomalous hump phenomenon turns more serious after inserting 5 ALD cycles of La_2_O_3_ passivation layer. While with the further increase of La_2_O_3_ passivation layer thickness, the humps of *C-V* curves are obviously reduced. Besides, as the thickness of La_2_O_3_ passivation layer increases, different degrees of frequency dispersion phenomenon at the weak inversion regions could be observed from the multi-frequency *C-V* curves in Figure 7, and the degrees of frequency dispersion phenomenon show a similar variation tendency with the anomalous humps in the *C-V* curves measured at 100 kHz. It has been reported that the existence of slow interface traps (*Q*_it_) should be responsible for the anomalous phenomenon at the weak inversion regions of *C-V* curves [26,27]. Considering this, the interface state density (*D*_it_) values of S1~S4 are discussed using the following relation of single-frequency approximation method through the forward swept *C-V* and *G-V* curves [28]:

(4) $$D_{\mathrm{it}}=\frac{2}{qA}\frac{\frac{G_{\max}}{\omega }}{\left[{\left(\frac{G_{\max}}{\omega C_{\mathrm{ox}}}\right)}^{2}+{\left(1-\frac{C_{\max}}{C_{\mathrm{ox}}}\right)}^{2}\right]}$$

where *q* is the elementary charge (1.602 × 10^−19^ C), *A* is the area of the top electrode, *C*_ox_ is the gate oxide capacitance as defined in Equation (1), *G*_max_ is the peak value of *G-V* curve, and *C*_max_ is the capacitance corresponding to *G*_max_ at the same gate applied voltage. Following Equation (4), the *D*_it_ values for the fabricated MIS capacitors using S1~S4 as insulators are calculated to be 9.18 × 10^12^, 9.72 × 10^1^^2^, 3.95 × 10^12^, and 2.71 × 10^12^ eV^−1^cm^−2^, respectively. The *D*_it_ value increases slightly after inserting 5 ALD cycles of La_2_O_3_ passivation layer, which is in consistent with the variation tendency of the humps in *C-V* curves. The increment of *D*_it_ is reported to be caused by an incomplete reaction of the precursor molecules during the first few ALD cycles [29], which brings in extra interface states at the interface. As the ALD cycles of La_2_O_3_ passivation layer increase, the improvement of the La_2_O_3_ passivation layer on the interfacial properties of gate insulators/Ge interfaces plays a more and more important role, contributing to the decrease of interface traps for the samples S3 and S4. For accuracy, the *D*_it_ values were also extracted using conductance method [30] measured from 1 kHz to 1 MHz, as shown in Figure 8. It could be observed that for each energy state, the *D*_it_ value extracted by conductance method increases slightly after inserting 5 ALD cycles of La_2_O_3_ passivation layer, while with the further increase of La_2_O_3_ passivation layer thickness, the *D*_it_ value shows decrease trend. This result is in good consistency with the single-frequency approximation method result. The contribution of the La_2_O_3_ interfacial passivation layer to the amount of interface traps at the La_2_O_3_/Ge interface could be explained as follows: during the ALD process of La(^i-^PrCp)_3_ and H_2_O precursors and the high temperature PDA process, La_2_O_3_ is more likely to react with outdiffused Ge atoms and Ge oxides nearby the interface to form stable LaGeO_x_ compound. The stable LaGeO_x_ compound could effectively suppress Ge outdiffusion, and further inhibit the decomposition or desorption from GeO_2_ to volatile GeO (following the reaction equation of GeO_2_ + Ge → 2GeO), contributing to the reduction of structural defects and dangling bonds [31,32]. The *D*_it_ value (2.71 × 10^12^ eV^−1^cm^−2^) of sample S4 achieved in our work is comparable with the results obtained in the reported literatures at La_2_O_3_/Ge interfaces of 3 × 10^12^ [8] and 3.5 × 10^12^ eV^−1^cm^−2^ [25], indicating that a La_2_O_3_ passivation layer with 15 ALD cycles (~1.3 nm) can effectively suppress the generation of interface states at high k dielectrics/Ge interfaces.

It is also observed from the *C-V* curves that the flat band voltages (*V*_FB_) translate toward positive voltages with the increase of La_2_O_3_ passivation layer deposition cycles. To further investigate this phenomenon in detail, the *V*_FB_ values of the capacitors were extracted using the Hauser NCSU CVC simulation software taking into account of quantum-mechanical effects [33]. Considering the work function difference between the top electrode aluminum and the n-type Ge substrate with a doping concentration of 1 × 10^16^ cm^−3^, the ideal *V*_FB_ should be 0.03 V. However, the actual *V*_FB_ swept forward for the control sample is −0.206 V. The negative *V*_FB_ shift indicates the existence of effective positive oxide charges in the HfO_2_ film. The effective positive oxide charges may be attributed to the existence of positive fixed oxide charges and oxide trapped charges mainly consisting of oxygen vacancies and structural defects in the gate insulators and/or nearby the insulators/Ge interface. Compared with the control sample, the forward swept *V*_FB_ values of MIS capacitors S2~S4 were extracted to be −0.083, −0.071, and −0.048 V, separately. The positive shifts of *V*_FB_ with the increase of La_2_O_3_ passivation layer thickness reveal the reduction of oxide charges after the Ge surface passivation treatment using La_2_O_3_ as the passivation layer. Considering this, the trapped charge density (*N*_ot_) is estimated using the midgap charge separation method through the *C-V* hysteresis characteristics following the Equation (5) [34]:

(5) $$N_{\mathrm{ot}}=\frac{\Delta V_{\mathrm{FB}}C_{\mathrm{ox}}}{qA}$$

where Δ*V*_FB_ is the hysteresis width of *V*_FB_, *C*_ox_ is the oxide capacitance, *q* is the elementary charge (1.602 × 10^−19^ C), and *A* is the area of the electrode. For the control sample, using the exacted Δ*V*_FB_ value of 685 mV, a trapped oxide charge density of 6.03 × 10^12^ cm^−2^ is obtained according to Equation (5). After the insertion of La_2_O_3_ passivation layer, the trapped oxide charge densities of samples S2, S3 and S4 are calculated to be 4.74 × 10^12^, 2.87 × 10^12^, and 1.79 × 10^12^ cm^−2^, respectively. These parameter results concerning the electrical properties are summarized in Table 1. As suspected, the trapped oxide charge density reduces with the increase of La_2_O_3_ thickness. We ascribe the decrease of trapped charges to the reduction of oxygen vacancies and structural defects in the gate stack region nearby the interface. At the HfO_2_/Ge interface, the outdiffused Ge atoms will react with GeO_2_ to form unstable GeO, which could bring in a large number of oxygen vacancies and structural defects. The LaGeO_x_ compound formed by La_2_O_3_ and Ge could effectively suppress the outdiffusion of Ge atoms and the desorption of volatile GeO, contributing to the reduction of *N*_ot_ values.

**Figure 9:**
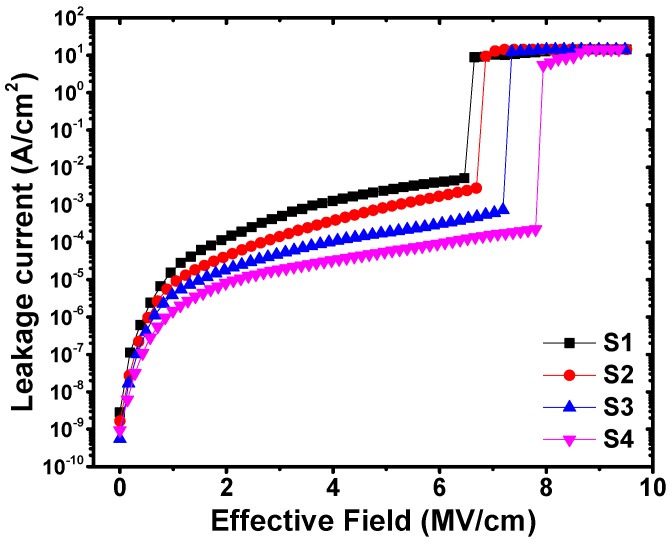
*J–V* characteristics of Al/HfO_2_/Ge and Al/HfO_2_/La_2_O_3_/Ge MIS capacitors.

Figure 9 shows the gate leakage current density as a function of the applied effective field for samples S1~S4. It is observed that the gate leakage current density of gate insulator/Ge structures obviously decreases with the increase of La_2_O_3_ layer thickness. At the applied effective field of 3 MV/cm, compared with the control sample, in the sample S4 the gate leakage current density decreases of almost an order of magnitude. The obvious decrease in the gate leakage current density could be ascribed to the reduction in the structural defects in the HfO_2_/La_2_O_3_ gate stack benefiting from the generation of stable LaGeO*_x_* component. Moreover, the gate leakage current density-voltage (*J-V*) characteristics show different breakdown behaviors, which suggests that the incorporation of La_2_O_3_ interfacial passivation layer improves the breakdown field strength of HfO_2_/Ge MIS capacitors. The improvement of the breakdown field strength is reported to be associated with a reduction in defects in gate dielectrics [35]. As mentioned above, the existence of La_2_O_3_ interfacial passivation layer could effectively suppress the generation of structural defects, oxygen vacancies and dangling bonds. The reduction of structural defects, oxygen vacancies and dangling bonds decreases the possibility to create a conduction path by forming a continuous chain connecting the gate to the semiconductor, resulting in higher breakdown field.

## 4. Conclusions

In summary, we investigate the effect of La_2_O_3_ passivation layer thickness on the interfacial and electrical properties of HfO_2_/Ge MIS structures. It is found that the gate leakage current density of the MIS capacitor after inserting 15 ALD cycles (~1.3 nm) of La_2_O_3_ passivation layer gains almost one order of magnitude decrease than that of the Al/HfO_2_/Ge MIS sample. Besides, a relatively low *D*_it_ value of 2.71 × 10^12^ eV^−1^cm^−2^ is achieved. The thickness of the La_2_O_3_ passivation layer also affects the dielectric constants, *V*_FB_ shifts, hysteresis behaviors and the shapes of dual-swept *C-V* curves. The improvement on the interfacial and electrical properties with the increase of La_2_O_3_ passivation layer deposition cycles is related to the formation of a stable LaGeO*_x_* layer on the Ge surface, which restrains the decomposition or desorption from GeO_2_ to volatile GeO, contributing to the decrease of structural defects. These results provide us with quite an effective method for realizing high-quality dielectric/Ge interfaces for Ge-based MIS devices.

## Figures and Tables

**Table 1:** The electrical parameters extracted from the fabricated MIS capacitors without and with La_2_O_3_ interfacial passivation layers.

Sample	*C*_ox_ (µF/cm^2^)	*k*	△*V*_FB_ (mV)	*D*_it_ (eV^−1^cm^−2^)	*N*_ot_ (cm^−2^)
S1	1.411	10.69	685	9.18 × 10^12^	6.03 × 10^12^
S2	1.510	13.43	504	9.72 × 10^1^^2^	4.74 × 10^12^
S3	1.712	15.97	269	3.95 × 10^12^	2.87 × 10^12^
S4	1.883	18.46	152	2.71 × 10^12^	1.79 × 10^12^

## Supplementary Materials

The following are available online at https://www.mdpi.com/2079-4991/9/5/697/s1, Table S1: Thickness of the deposited films (including interfacial layer) on Ge substrates.
