# Supplementary material for: Electrical Properties and Interfacial Issues of HfO2/Ge MIS Capacitors Characterized by the Thickness of La2O3 Interlayer
Source: Nanomaterials (Basel). 2019 May 4;9(5):697. doi: 10.3390/nano9050697 (PMC6567279; doi:10.3390/nano9050697)
Supplement: Supplementary file 1 [file nanomaterials-09-00697-s001.pdf]

Supplementary Materials

Electrical properties and interfacial issues of HfO_2/Ge MIS capacitors characterized by the thickness of La_2O_3 interlayer

Lu Zhao, Hongxia Liu *, Xing Wang, Yongte Wang and Shulong Wang

Key Laboratory for Wide Band Gap Semiconductor Materials and Devices of Education, School of Microelectronics, Xidian University, Xi'an 710071, China; lzhaoxd@163.com (L.Z.); xwangsme@xidian.edu.cn (X. W.); mikewyt@163.com (Y.W.); slwang@xidian.edu.cn (S.W.)

* Correspondence: hxliu@mail.xidian.edu.cn (H.L.); Tel.: 86-29-88204085 (H. L.)

In this manuscript, the thickness of the deposited dielectric films was obtained by averaging the spectroscopic ellipsometry testing values of different positions on Ge substrates. In order to evaluate the discrete degree of thickness testing data, the corresponding 95% confidence interval for the average thickness of the deposited films was calculated as shown in Table 1. It turns out that the confidence intervals for all of the samples are relatively narrow, indicating the average thickness values are credible and representative to show the average thickness level of the deposited films.

Table S1 Thickness of the deposited films (including interfacial layer) on Ge substrates.

Sample	Average thickness (nm)	95% Confidence interval (nm)
S1	6.71	(6.661, 6.759)
S2	7.89	(7.854, 7.926)
S3	8.26	(8.235, 8.285)
S4	8.95	(8.929, 8.971)
